# Comparative chloroplast genomes and phylogenetic analyses of *Pinellia*

**DOI:** 10.1007/s11033-022-07617-5

**Published:** 2022-06-11

**Authors:** Ning Cui, Weixu Chen, Xiwen Li, Ping Wang

**Affiliations:** 1 Central Laboratory, Shandong Academy of Chinese Medicine, Ji’nan, China; 2 Shang Yao Hua Yu (LinYi) Traditional Chinese Medicine Resources Co., Ltd, Linyi, China; 3 Institute of Chinese Materia Medica, China Academy of Chinese Medical Sciences, Beijing, China

**Keywords:** *Pinellia*, Phylogeny, Evolution, Divergence time estimation, Chloroplast genome

## Abstract

**Background:**

*Pinellia* Tenore (Araceae) is a genus of perennial herbaceous plants, all of which have medicinal value. The chloroplast (cp) genome data of *Pinellia* are scarce, and the phylogenetic relationship and gene evolution remain unclear.

**Methods and results:**

We sequenced and annotated the *Pinellia pedatisecta* cp genome and combined it with previously published genomes for other *Pinellia* species. We used bioinformatics methods to analyse the genomic structure, repetitive sequences, interspecific variation, divergence hotspots, phylogenetic relationships, divergence time estimation and selective pressure of four *Pinellia* plastomes. Results showed that the cp genomes of *Pinellia* varied in length between 168,178 (*P. pedatisecta* MN046890) and 164,013 bp (*P. ternata* KR270823). A total of 68–111 SSR loci were identified as candidate molecular markers for further genetic diversity study. Eight mutational hotspot regions were determined, including *psbI-trnG-UCC, psbM-rpoB, ndhJ-trnT-UGU, trnP-UGG-trnW-CCA, ndhF-trnN-GUU, ndhG-ndhE, ycf1-rps15* and *trnR-ycf1*. Gene selection pressure suggested that four genes were subjected to positive selection. Phylogenetic inferences based on the complete cp genomes revealed a sister relationship between *Pinellia* and *Arisaema* plants whose divergence was estimated to occur around 22.48 million years ago. All *Pinellia* species formed a monophyletic evolutionary clade in which *P. peltata*, rather than *P. pedatisecta*, earlier diverged, indicating that *P. pedatisecta* is not the basal taxon of *Pinellia* but *P. peltata* may be.

**Conclusions:**

The cp genomes of *Pinellia* will provide valuable information for species classification, identification, molecular breeding and evolutionary exploration of the genus *Pinellia*.

**Supplementary Information:**

The online version of this article (10.1007/s11033-022-07617-5) contains supplementary material, which is available to authorized users.

## Introduction

*Pinellia* Tenore is a small eastern Asian genus in the Araceae family. Although there are only seven perennial herbaceous species in *Pinellia* genus [1], every member it contains is important traditional Chinese medicinal plant and was recorded in Chinese herb classics more than 2000 years ago; the most famous among them is *P. ternata*, with an annual demand of 5500–6000 tons [2]. They have been used for the treatment of viper bites, lumbago, allergic reaction and externally to treat traumatic injury, abscesses, neck lymphosarcoma, breast mastitis and uterine cancer [3, 4].

In recent years, the phylogeny and evolution of monocots have come under intense scrutiny with the rapid development of molecular phylogenetic systematics, and multiple studies have highlighted the phylogeny of *Pinellia* as problematic [5, 6]. The phylogenetic position of the *Pinellia* genus in Araceae has been controversial, and sister groups of *Pinellia* show discrepancy under different classification systems and studies [7, 8]. The six major taxonomic systems of Araceae, namely, Schott system [9], Engler system [10], Hutchinson system [11], Grayum system [12], Bogner and Nicolson system [13] and Mayo et al. system [14], have different views on the sister genus of *Pinellia*, with four genera (*Crytocoryne*, *Langenandra*, *Ambrosina* and *Arisaema*) as candidates. With the development of molecular systematics, analyses of the plastome gene and restriction-site sequences suggested that the *Arisaema* genus is strongly related to *Pinellia* and is the sister genus of *Pinellia* [7, 15, 16]. Contrary to the above classification, Keating [17] and Bogner and Petersen [18] based on morpho-anatomical data argued that *Arisaema* and *Pinellia* cannot gather into a unique clade for their morphological discrepancy of stamens.

Members of *Pinellia* exhibit wide variations in flower, leaf, bulblet, spathe and ovule characteristics [19, 20]. The low-level taxonomy and interspecific phylogeny of *Pinellia* are difficult to address based on morphological traits. The most comprehensive below-genus phylogenetic analysis to date has been provided by Yin, a study using a matrix of all seven *Pinellia* species and ITS and *trnL-F* DNA barcode sequences [21]. Combined with the morphological traits, Yin concluded that *P. pedatisecta* is the basal taxon of *Pinellia* and suggested that its taxonomic rank should be elevated to a section. However, our group previously conducted interspecific phylogenetic analyses of *Pinellia* by using four DNA barcode sequences (*ITS, matK, rbcL* and *trnL-F*) with the same method as Yin. The topologies of four barcodes were not consistent, with *P. pedatisecta* in the outer layer of ITS and *matK* trees and in the inner layer of *rcbL* and *trnL-F* trees, receiving considerably lower bootstrap values (Supplementary Fig. S1) probably due to insufficient sequence length and interspecific variations.

The chloroplast (cp) is an important self-replicating organelle that plays a crucial role in photosynthesis and in the synthesis of pigment, protein and starch [22]. The cp contains its own circular double-stranded genome, which is inherited maternally in most angiosperms or paternally in some gymnosperms [23]. Unlike the nuclear genome, the cp lacks meiotic recombination. These properties, along with adequate levels of polymorphism, make it a suitable molecule for studies on phylogeny and evolution [24]. Scientists, especially Henriquez CL and Abdullah research group, have long been devoted to the plastome phylogeny of the Araceae family [25–28], and sequenced the cp genome of *P. pedatisecta* (MN046890) in 2020 [22]. Nevertheless, their studies focused more on the backbone phylogeny at the taxonomic level of the entire or subfamilies of Araceae, and local phylogenetic relationships of *Pinellia* genus were rarely paid attention to. Moreover, we previously performed a comparative genomics analysis within *Pinellia* genus, and found that the published *P. pedatisecta* cp genome was relatively different from those of two other species, *P. peltata* (NC052862) [29] and *P. ternata* (KR270823) [30] in sequence length, gene content, GC content, etc. We determined to identify, sample and sequence the cp genome of *P. pedatisecta* independently for a reliable phylogeny of *Pinellia* species. The plastome evolution of *Pinellia* was also discussed, including the estimations of gene selection pressure and divergence time which have not been studied before.

To reveal the interspecific diversification pattern of *Pinellia*, we determined the complete cp genome of *P. pedatisecta* and compared its sequence features with three other *Pinellia* plastomes. The main goals of this study were to (1) characterize and compare the cp genomes of *Pinellia* and detect the sequence differences between *Pinellia* species and between published and newly assembled cp genomes of *P. pedatisecta*; (2) identify simple sequence repeats (SSRs), long repeats and genetically variable regions and select divergence hotspots as candidate DNA barcodes; (3) reconstruct phylogenetic relationships of *Pinellia* species based on the cp genome alignments and verify their phylogenetic position within Araceae; and (4) estimate genes selection pressure and divergence time for determining the relative order and spacing of speciation events of *Pinellia*. Comparative cp genomic analysis could provide theoretical basis to further understand the evolution of the Araceae family and additional insights into the long-standing controversial intergenus and intragenus phylogeny of *Pinellia*.

## Materials and methods

### Plant material, DNA extraction, sequencing, assembly and annotation

In our study, the cp genome of *P. pedatisecta* was sequenced to explore the phylogeny and evolution of *Pinellia*. Fresh leaves of *P. pedatisecta* from Linyi City, Shandong Province were sampled. Voucher specimens were deposited in Shandong Academy of Chinese Medicine. Total genomic DNA was extracted from 100 mg of silica-dried leaf by using a DNeasy Plant MiniKit (Qiagen, CA, USA) according to the manufacturer’s instructions [31]. The quantity and quality of genomic DNA were examined by using ND-2000 spectrometer (ThermoFisher Scientific, Wilmington, DE, USA) and 0.8% agarose gel electrophoresis. The chloroplast genome of *Salvia plebeia* (NC050929) was assembled in our previous study [31]. Taking this work as a guidance, the DNA sample pre-treatment (Covaris M220 [Covaris, US; 250 bp] and VAHTS™ Universal DNA Library Prep Kit [Vazyme, China]), whole genome sequencing (Illumina Hiseq 1500 platform [Illumina Inc., USA]), cp genome assembly (Skewer, Basic Local Alignment Search Tool [BLAST], SOAPdenovo v.2.04, GapCloser and MUMmer), junction validation, and annotation (Plann v. 1.1.2, BLAST and Apollo) were performed in turn with the cp genome of *P. peltata* (NC052862) as a reference sequence. The primers for junction validation are listed in Supplementary Table S1. The cp genome obtained in this study has been submitted to the NCBI database (www.ncbi.nlm.nih.gov). The physical map of *P. pedatisecta* cp genome was produced with Chloroplot (https://irscope.shinyapps.io/Chloroplot/).

### Repeat structure identification

SSRs were identified by MISA [32], and the minimum thresholds for mono-, di-, tri-, tetra-, penta- and hexa-nucleotides were set to 10, 6, 5, 5, 5 and 5, respectively. REPuter [33] was used to detect two kinds of long repeats: forward and palindromic repeats which have been reported relatively more prevalent in the cp genomes of Araceae family [22, 26]. Detection parameter settings were used as follows: repfind -d -p -h 3 -l 30 -best 50. Tandem Repeats Finder (http://tandem.bu.edu/trf/trf.html) was used to find tandem repeats with the default settings.

### Genome comparative analysis

In addition to the newly sequenced cp genome, 26 available cp genome sequences of *Pinellia* and related species were downloaded from the NCBI database: *P. pedatisecta* (MN046890), *P. ternata* (KR270823), *P. peltata* (NC052862), *Arisaema franchetianum* (MN046885), *Arisaema ringens* (MK111107), *Arisaema erubescens* (MT676834), *Arisaema nepenthoides* (MW338731), *Typhonium blumei* (NC051872), *Sauromatum giganteum* (NC050648), *Alocasia navicularis* (MN046882), *Colocasia esculenta* (JN105689), *Pistia stratiotes* (MN885890), *Amorphophallus konjac* (MK611803), *Calla palustris* (MN046887), *Epipremnum aureum* (KR872391), *Epipremnum amplissimum* (MN477424), *Aglaonema costatum* (MN046881), *Monstera adansonii* (MN046888), *Zantedeschia aethiopica* (KY792991), *Pothos scandens* (MN046891), *Symplocarpus renifolius* (KY039276), *Symplocarpus nipponicus* (MK341566), *Lemna minor* (DQ400350), *Acorus calamus* (AJ879453), *Acorus americanus* (EU273602) and *Acorus gramineus* (KP099646). The multiple sequence alignment of the 27 cp genome sequences was performed using MAFFT v.7 with the default settings and adjusted manually where necessary with BioEdit v.7.2.5 software. On the basis of the aligned sequence matrix of the cp genomes of *Pinellia*, interspecific nucleotide diversity (K) was evaluated by sliding window analysis with a step size of 1000 bp and window length of 2000 bp in DnaSP v.5.10 [34]. The evolutionary divergences of the four *Pinellia* cp genomes were evaluated using nucleotide differences and p-distance by MEGA v.10.0.4. The protein-coding sequences (CDSs) were extracted using Geneious v.2019.1.3 [35].

### Phylogenetic analyses

Two datasets (cp genome and coding sequence) were used to construct the phylogenetic topology of 27 *Pinellia* and related species with maximum likelihood (ML) methods, respectively. ML analyses were performed using RAxML 8.2.9 under the GTRGAMMA model with 1000 rapid bootstrap replicates. Three species of *Acorus* (*A. calamus*, *A. americanus* and *A. gramineus*) were used as outgroups.

### Divergence time estimation

Accurate estimation of the divergence time in a taxon is important to understand its evolutionary history. Divergence times were estimated using PAML mcmctree (PAML v.4.9j) [36] with the approximate likelihood calculation method. The analysis was performed on 27 complete cp genome sequences used in the phylogenetic analysis with three known calibration times: (1) divergence between *P. pedatisecta* and *T. blumei* was 36–32 million years ago (Mya); (2) divergence between *A. navicularis* and *P. stratiotes* was 85–46 Mya; and (3) all species except the genus *Acorus* from the Araceae family arose 122–117 Mya inferred from the published knowledge-based TimeTree (timetree.org). Posterior distributions of parameters were approximated using two independent mcmctree analyses of 10,000,000 generations with 20% burn-in. Tracer v.1.4.1 was used for checking the convergence of the chains through adequate effective sample sizes (ESSs).

### Gene selection site analysis

A total of 74 single-copy protein-coding genes shared by four *Pinellia* plastid genomes (Table 1) were extracted and aligned by Geneious v.9.0.5 [35] and MAFFT v.7. The ML tree was constructed using RAxML V.8.2.9 with the GTRGAMMA model based on complete cp genomes. Protein-coding exon and each value of *dN*, *dS* and *ω* were calculated using the site-specific model in the Codeml program (seqtype = 1, model = 0, Nsites = 0, 1, 2, 3, 7, 8) of PAML v.4.9j [36]. To determine the selected sites, we compared the model M0 (one ratio) versus M3 (discrete), M1 (neutral) versus M2 (positive selection) and M7 (beta) versus M8 (beta and ω) and carried out the three likelihood ratio tests (LRTs). Only consistent sites of positive selection with significant support from posterior probability (*p* of (*ω* > 1) ≥ 0.99; Bayes Empirical Bayes approach [BEB]) were identified. BEB recognized by Models M2 and M8 were further considered.

**Table 1:** Summary of features of four *Pinellia* chloroplast genomes

Taxon	Accession number	Length (bp)				Number of genes				GC content (%)			
		Genome	LSC	SSC	IR	Total	Protein coding	tRNA	rRNA	Genome	LSC	SSC	IR
*P. pedatisecta*	MZ702636	164,682	90,947	22,523	25,606	129	85	36	8	35.71	33.92	29.43	42.09
*P. pedatisecta*	MN046890	168,178	92,963	23,981	25,617	130	85	37	8	35.08	33.35	26.85	42.09
*P. peltata*	NC052862	164,923	90,089	24,871	24,981	130	86	36	8	36.51	34.53	31.77	42.44
*P. ternata*	KR270823	164,013	89,783	22,980	25,625	131	86	37	8	36.66	34.6	31.66	42.53

## Results

### Chloroplast genome features in *Pinellia*

**Fig. 1:**
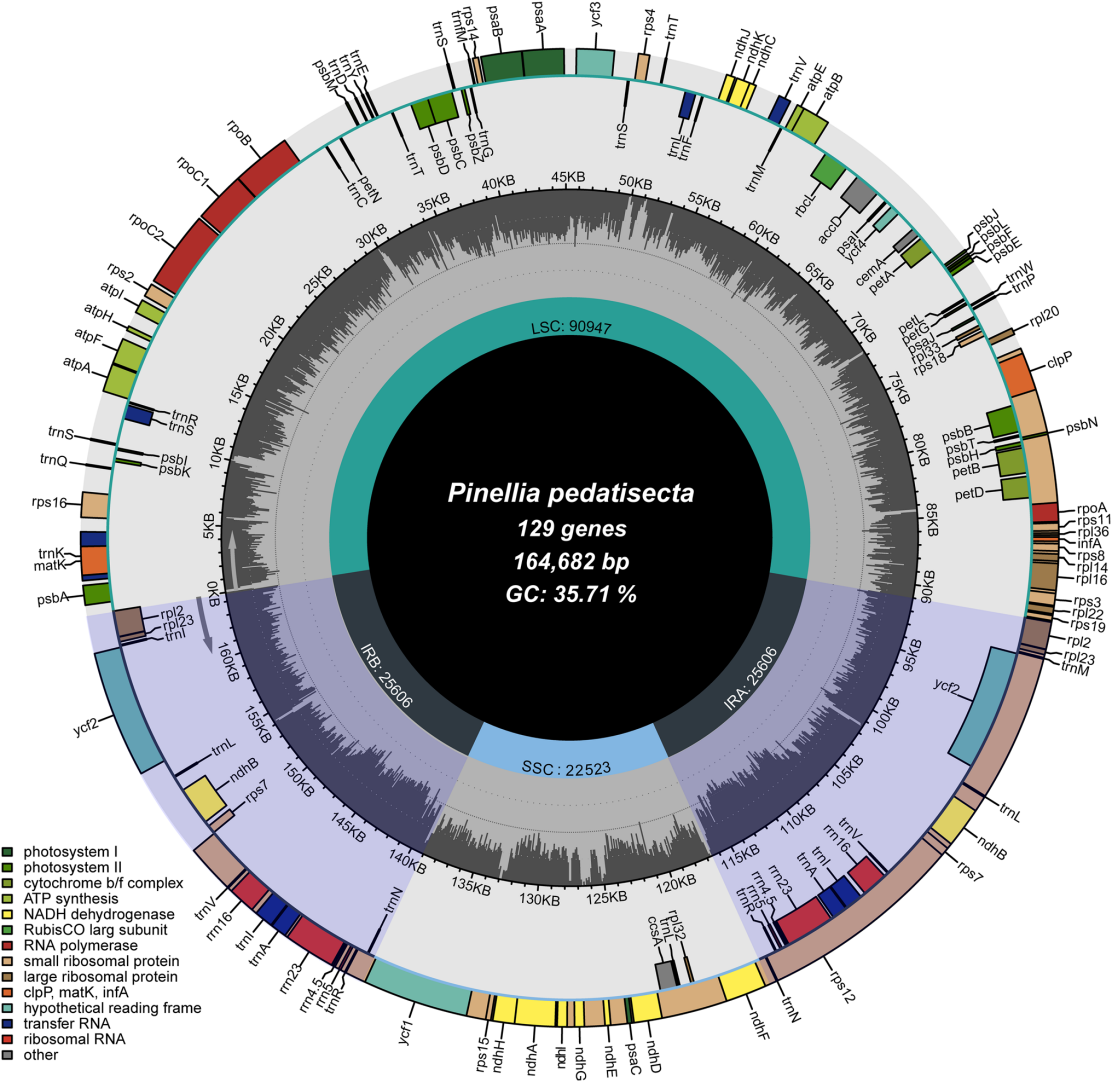
Chloroplast genome map of *P. pedatisecta* assembled in this study. The centre of the figure provides length information of the cp genome. In the first inner circle, the proportion of the shaded parts represents the GC content of each part. The gene names are labelled on the outermost layer. The transcription directions for the inner and outer genes are listed clockwise and anticlockwise, respectively

In our study, the cp genome of *P. pedatisecta* was sequenced, assembled and validated (Fig. S2). A total of 49,904,104 PE raw reads were generated using the Illumina Sequencing System. The novel cp genome sequence has been preserved in GenBank (MZ702636, Table 1). The cp genome of *P. pedatisecta* was circular double-stranded DNA and displayed a quadripartite structure (Fig. 1). The cp genome assembled in this study was 164,682 bp in length, which was 3,496 bp shorter than the published one (MN046890, 168,178 bp) mainly due to the contraction of the LSC region. Moreover, the GC content in the SSC region of the cp genome MZ702636 was 2.58% higher than that of MN046890. The cp genome MN046890 had the same rRNA gene content as that of MZ702636 but contained one more tRNA gene (*tRNA-His*) (Supplementary Table S2).

After downloading from the NCBI database, protein-coding gene number variations of 24 cp genomes of the Araceae family, including four *Pinellia* cp genomes, were analysed (Fig. S3). Compared with the cp genome of the model plant *Arabidopsis thaliana*, two genes, *infA* and *ycf68*, were inserted among most Araceae plants, while one-copy *ycf1* gene was missing. In the Araceae family, there were significant differences in the number of *ycf68* genes among species. The *ycf68* genes in 60% of Araceae cp genomes were completely missing, while the remaining 40% of the cp genomes had two copies. Even within the *Pinellia* genus, the number of *ycf68* genes was also inconsistent. Nevertheless, there was no discrepancy in the type and number of protein-coding genes between our newly assembled cp genome (MZ702636) and the published one (MN046890).

### SSR and repeat sequence analyses

**Fig. 2:**
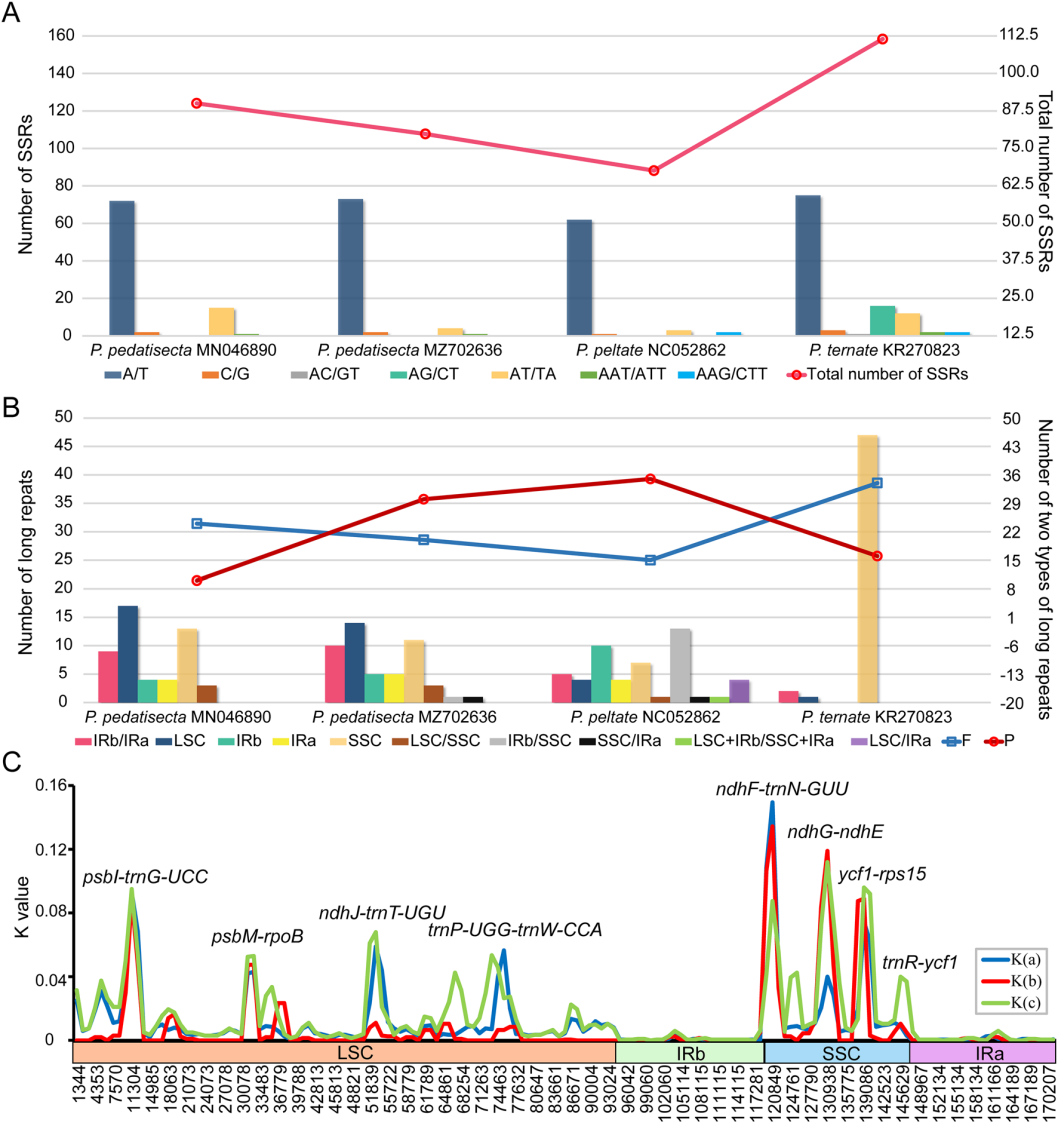
The SSRs (**A**), interspersed repeated sequences (**B**), and interspecific nucleotide diversity (**C**) among the chloroplast genomes of *Pinellia*. X-axis in Fig. 3C: position of a window. Y-axis in Fig. 3C: sequence divergence (K values) between species of each window. K(a): K values between *P. pedatisecta* and *P. peltata*; K(b): K values between *P. pedatisecta* and *P. ternata*; K(c): K values between *P. ternata* and *P. peltata*

The number of SSRs in the four *Pinellia* cp genomes ranged from 68 (*P. peltata*) to 111 (*P. ternata*, Fig. 2A and Supplementary Table S3). Three kinds of SSRs were discovered, namely, mononucleotide, dinucleotide and trinucleotide. Among each *Pinellia* species, mononucleotide repeats were the most common, whereas trinucleotide repeats accounted for the lowest proportion of SSRs. The number of A/T mononucleotide repeats exceeded that of the other three types combined (Fig. 2A). Interspersed repeated sequences were identified by using REPuter for four plastomes (Fig. 2B and Supplementary Table S4). Except for IR regions, the repeats’ length ranged from 31 to 328 bp with forward (F) repeats as the relative prevalent type. Most interspersed repeats of two *P. pedatisecta* cp genomes were located in the LSC regions, while the long repeats of *P. peltata* and *P. ternata* were predominantly in the IRb/SSC and SSC regions, respectively. The total number of tandem repeats for four plastid genomes was in the range of 34–274. *P. peltata* had the least repeats, and *P. pedatisecta* (MN046890) had the most (Supplementary Table S5).

### Comparative genomic analyses

The differences and evolutionary divergences among four *Pinellia* cp genomes were compared using nucleotide substitutions and sequence distance (Supplementary Table S6). Across all four *Pinellia* cp genomes, the p-distance was 0.000402–0.013668, and the value of nucleotide differences was 66–2459. The comparison results of nucleotide substitution and genetic distance were consistent. The sequence divergence level of two cp genomes of *P. pedatisecta* was the lowest. The differentiation between *P. pedatisecta* and *P. ternata* was less than that between *P. pedatisecta* and *P. peltata*. Among three *Pinellia* species, *P. ternata* and *P. peltata* were the most genetically distant. Moreover, the K value (sequence divergence between species) was calculated, and the sliding windows of the K values were constructed by DnaSP (Fig. 2C and Supplementary Table S7). Figure 2C showed that the sequence divergence between *P. pedatisecta* and *P. ternata* was much lower than the two other K values. The sequence divergence between *P. pedatisecta* and *P. peltata* was not so different from that between *P. ternata* and *P. peltata*. Eight highly variable regions with great K values were detected, namely, *psbI-trnG-UCC, psbM-rpoB, ndhJ-trnT-UGU, trnP-UGG-trnW-CCA, ndhF-trnN-GUU, ndhG-ndhE, ycf1-rps15* and *trnR-ycf1*. Four of these regions were located in the LSC region, and the remaining four were located in the SSC region, all of which were present in the non-coding regions.

### Phylogenetic analyses

**Fig. 3:**
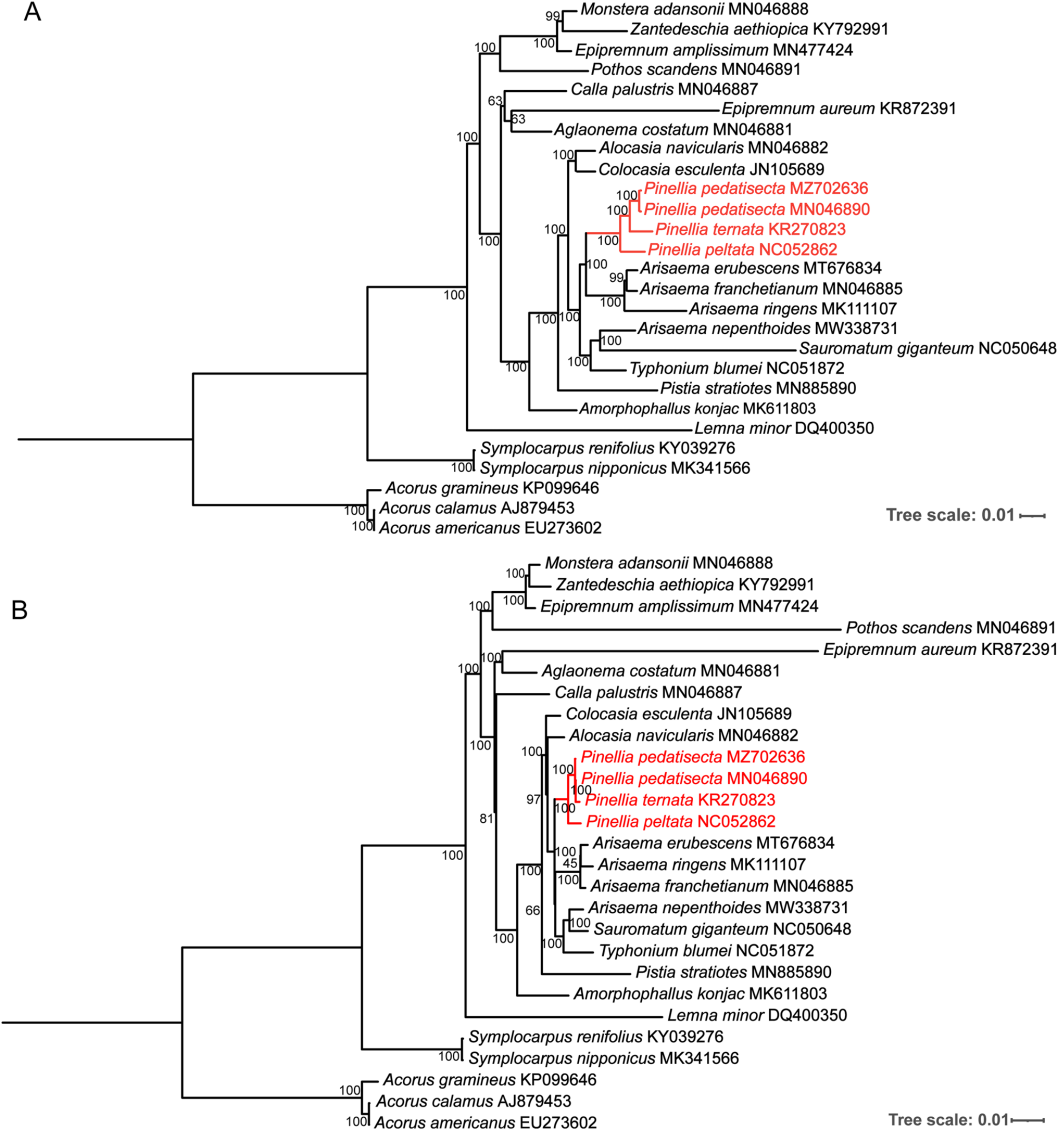
Phylogenetic trees constructed with the whole cp genomes (**A**) and their protein-coding sequences (**B**) by using ML method. Numbers near each branch are bootstrap values

To analyze the phylogenetic relationship of *Pinellia* species, we constructed phylogenetic trees using whole cp genome sequences and their CDSs by ML methods. For *Pinellia* species, the topological structure obtained by either complete cp genome or CDSs was roughly identical (Fig. 3). The topology based on entire cp genomes showed that *Pinellia* species were monophyletic and clustered into three clades: one for two *P. pedatisecta* plastomes, one for *P. ternata* and one for *P. peltata* with strong support (bootstrap value 100%). *P. pedatisecta* and *P. ternata* clustered together, exhibiting the highest genetic similarity among the studied representatives of *Pinellia* genus. In contrast to the CDSs phylogeny (Fig. 3B), in which *Pinellia* species were placed most closely to *Arisaema*, *Sauromatum* and *Typhonium* clade, the whole-length plastome phylogeny placed some *Arisaema* species as sister to the genus *Pinellia* with 100% bootstrap support (Fig. 3A).

### Divergence time estimation

In this study, we used full-length sequences of cp genomes of 23 Araceae family plants (including four *Pinellia* cp genomes) and three outgroups to estimate the divergence times of major clades in the Araceae family. Our dating analysis resulted in estimates for the crown node of the Araceae family of 91.99 Mya (95% highest posterior density [HPD] = 46.69–125.17 Mya; node 1 in Fig. S4; Supplementary Table S8) in the early Cretaceous. The divergence time of the four genera *Pinellia, Arisaema*, *Sauromatum* and *Typhonium* was estimated at 25.65 Mya (95% HPD = 12.93–36.35 Mya; node 2) in the Oligocene. The age estimation for the crown node of *Pinellia* and most species of *Arisaema* was dated to 22.48 Mya (95% HPD = 11.39–33.32 Mya; node 3) in the late Oligocene and early Miocene. *Pinellia* diverged from its sister clade at 13.09 Mya (95% HPD = 4.91–21.96 Mya; node 4 in Fig. S4; Supplementary Table S8) in the Miocene.

### Gene selection pressure analysis of protein sequences

The site-specific selective pressure on four *Pinellia* plastomes was assumed using the site model in PAML program. Three pairs of site model comparisons (M0 vs M3, M1 vs M2a and M7 vs M8) showed four protein-coding genes subjected to positive selection (LRT of the three comparisons all *p* < 0.05; Supplementary Tables S9 and S10), namely, *petB*, *rpoC1*, *rps12* and *ycf1*. Of these genes, *ycf1* had the highest number of sites (21 sites; Table S9), followed by *rpoC1* (4 sites), *petB* (1 site) and *rps12* (1 site).

## Discussion

### Phylogenetic position of the *Pinellia* genus in Araceae

In the previous classification systems, the *Pinellia* genus belonged to the Araceae family, but its sister genus remains controversial. In the Schott system [9], which is the first classification system of the Araceae family, the genus *Pinellia* was placed in the tribus Alleluehieae, related to two genera of *Crytocoryne* and *Langenandra*. In the Engler system [10], *Pinellia* belonged to the subtrib. Pinelliinae, trib. Areae, subfam. Aroideae, in the same tribus as the *Arisaema* genus. In the Hutchinson system [11], this genus was placed in trib. Areae, related to two genera of *Crytocoryne* and *Ambrosina*. In the classification system of Grayum [12], *Pinellia* belonged to trib. Pinellia, subfam. Aroideae. After modifying the Engler system by Bogner and Nicolson [13], the *Pinellia* genus changed to the subtrib. Atherurinae, trib. Pinellia, subfam. Aroideae. Mayo et al. [14] conducted taxonomic analysis of 106 taxa in the Araceae family using 63 morphological and anatomical traits and proposed the latest classification system of the Araceae family. In this system, *Pinellia* was placed in trib. Arisaemateae, subfam. Aroideae with the *Arisaema* genus as sister group. Since the 21st century, with the development of molecular systematics, many studies have investigated the phylogenetic position of the *Pinellia* genus in Araceae [7, 15–18], yet controversy regarding molecular systematics and morphological classification persists. French et al. [16], Cabrera et al. [7] and Cusimano et al. [15] performed molecular phylogenetic analyses by applying different types and numbers of DNA marker sequence data, and they discovered that the *Arisaema* genus is strongly related to *Pinellia* among Araceae plants, and is the sister genus of *Pinellia*. Contrary to the above classification, Keating [17] and Bogner and Petersen [18] based on morpho-anatomical data argued that *Arisaema* and *Pinellia* cannot be grouped into a unique clade. One significant morphological discrepancy between them was that almost all *Arisaema* species have at least partially fused stamens, whereas *Pinellia* and other related genera (e.g. *Sauromatum* and *Typhonium*) have free stamens.

The cp genome is one of three subcellular compartments in the plant genome, and it is mainly inherited from the maternal parent [37]. Given its high conservation and abundant interspecific variation, the cp genome has the potential to provide distinguishing differences that can help molecularly classify closely related species [38]. With advances in high-throughput sequencing, achieving the cp genome is easily acquirable at a large scale with low costs. Researchers have proposed the entire cp genome as a super barcode to discriminate and classify closely related species [39]. To date, phylogenies of several genus-level taxa have been further clarified by using cp genome sequences, such as *Epimedium* [40]*, Paris* [41] and *Sanguisorba* [42]. Hence, we compared 24 cp genomes of the Araceae family, including four plastomes of *Pinellia*, to explore the cp genome molecular phylogeny of *Pinellia*. The resulting phylogeny based on entire cp genomes in this study showed that *Pinellia* and *Arisaema* plants were gathered into one branch and sister groups to each other with a well-supported bootstrap value (100%; Fig. 3A). Although the Araceae family is a major group of monocotyledons, there is still a limited number of cp genomes available from Araceae species, which may result in some congeneric species, such as *Arisaema* plants, not clustering together in one phylogenetic branch (Fig. 3). The absence of plastid genomes from three other candidates of the *Pinellia* sister groups*, Crytocoryne*, *Langenandra* and *Ambrosina* [21], could also hinder our discovery of the intergenus phylogeny of *Pinellia*.

### Phylogeny within the *Pinellia* genus

Despite recent advances in molecular phylogenetic studies, deep evolutionary relationships and below-genus taxonomic classification of *Pinellia* remain unresolved. Yin [21] made the first comprehensive phylogenetic analyses of all seven *Pinellia* species by using the sequences of ITS and *trnL-F* DNA barcodes. The resulting phylogeny of both ITS and *trnL-F* sequences supported that *P. pedatisecta* and the six remaining *Pinellia* species were sister species, and *P. pedatisecta* served as a basal taxon in *Pinellia*. Moon et al. [43] reconstructed the phylogenetic trees of three species, *P. ternata, P. tripartita* and *P. pedatisecta*, with *matK* and *rbcL* sequences; they found that the topology of two trees is inconsistent. The basal taxon in the *matK* tree was *P. pedatisecta*, congruent with that by Yin [21], while the basal taxon in the *rbcL* tree was *P. ternata.* Furthermore, after morphological investigation, Yin found that *P. pedatisecta* has many characteristics of the *Pinellia* genus. These traits include perennial herb with a small tuber at the top of the main tuber, persistent spathe, female inflorescence adnate to the spathe, unisexual flowers, no perianth, one straight ovule and green fruit. Hence, combined with the molecular phylogeny, Yin concluded that the basal taxon of the *Pinellia* genus is *P. pedatisecta*, the species native to shady woodland areas, forested slopes and valleys in northern and western China; he suggested that the taxonomic rank of *P. pedatisecta* should be elevated to a section of *Pinellia* [21], which was the first and only below-genus taxonomic recommendation of *Pinellia*.

In our opinion, *P. pedatisecta* indeed has some of the characteristic traits of *Pinellia*, but it also possesses so many characteristics significantly different from other congeneric species. For example, lanceolate spathe, no constriction in the tube and eaves of spathe, no diaphragm between the female inflorescence and male inflorescence and no bulblet on the petiole [15, 19, 20]. Our group previously conducted phylogenetic analyses of *Pinellia* by using four different barcode sequences with maximum parsimony (MP) method. The topologies of the four barcodes were not consistent, with poor bootstrap values (Fig. S1) probably due to insufficient sequence length and interspecific variation. Hence, plastid genomes, an ideal model for evolutionary and comparative genomic studies of related species, were necessary here for a molecular phylogeny of *Pinellia* with a significantly higher resolution.

After comparing the structural organization of three previously published cp genomes of *Pinellia*, we found that the sequence similarity between the published *P. pedatisecta* plastome (MN046890) and the cp genomes of *P. peltata* (NC052862) and *P. ternata* (KR270823) was clearly lower than expected in the following five aspects.

1. The length of the *P. pedatisecta* plastome was significantly longer than that of the two other species (Table 1), specifically 3.2 kb longer than that of *P. peltata* and even 4.1 kb longer than that of *P. ternata*. The length of the LSC region in *P. pedatisecta* was 3.18 kb longer than that of *P. ternata*.
2. With regard to protein-coding gene content, all two *ycf68* genes were missing in the published cp genome of *P. pedatisecta* (Fig. S3), compared with *P. peltata* and *P. ternata*, which could have the potential to affect the physiological functions of plants, although some authors suggested that the *ycf68* gene likely does not encode a protein [44].
3. The GC content of published *P. pedatisecta* was lower than that in *P. peltata* and *P. ternata* (Table 1), especially in the SSC region, which was 4.92% lower than that in *P. peltata* and 4.81% lower than that in *P. ternata*.
4. After the alignment of three published cp genomes and further estimation of locally collinear blocks (LCBs) with MAUVE 2.4.0, the gene order comparison revealed one rearrangement (~ 128 bp) between the published plastomes of *P. pedatisecta* and *P. ternata* (Fig. S5A and S5B).
5. A significant insertion/deletion variation was noted across three cp genomes in this group, located between *tRNA-Ser (GCU)* and *tRNA-Ser (CGA)* genes in the LSC region with the length of ~ 1.2 kb (10,714–11,950 bp in the published *P. pedatisecta* cp genome and 10,192–10,234 bp in the self-assembled one; Fig. S5C and S5D).

These five sequence discrepancies do not follow the rule of “chloroplast genomes of related species, especially those within the same genus, are generally highly conserved” [45, 46]. Therefore, we determined to identify, sample and sequence the plastome of *P. pedatisecta* independently for further phylogenetic analyses of *Pinellia*.

In this study, the phylogenetic tree based on whole cp genomes showed that the species of *Pinellia* were clustered into three clades: one for two *P. pedatisecta* plastomes, one for *P. ternata* and one for *P. peltata* with high support values (Fig. 3). *P. pedatisecta* and *P. ternata*, which possess pedate and three full-lobed leaves, respectively, grouped together in topology, exhibiting the highest genetic similarity among the studied representatives of *Pinellia* genus. In contrast, *P. peltata*, characterized by undivided and peltata leaf and development of stem tuber, was placed in the outer layer of *Pinellia* topology. *P. pedatisecta* is buried inside the cp genome phylogeny, incongruent with the phylogeny from ITS and *trnL-F* sequences by Yin [21]. Furthermore, comparative genomic analyses showed that the nucleotide substitutions and sequence distance between *P. pedatisecta* and *P. ternata* were the smallest (1275 and 0.007206; Table S6), while that between *P. ternata* and *P. peltata* was the largest (2459 and 0.013668). The interspecific nucleotide diversity (K value) between *P. pedatisecta* and *P. ternata* was considerably lower than the two other K values (Fig. 2C), both of which confirmed that *P. peltata* shared less sequence similarity with *P. pedatisecta* and *P. ternata*. Limited by the number of *Pinellia* cp genomes published so far, we cannot determine whether *P. peltata* is the ancestor of *Pinellia* species, but it is possible. *P. peltata* has the same floral morphological characteristics as other *Pinellia* species, except for *P. pedatisecta* [29]. For example, solitary inflorescence, persistent spathe, with constriction in the tube and eaves of spathe, female inflorescence adnate to the spathe, with diaphragm between the female inflorescence and male inflorescence, unisexual flowers, no perianth, one locular ovary and one straight ovule [21]. However, the peltata leaf characteristics of *P. peltata* are considerably different. Thus, the below-genus taxonomic classification of *Pinellia* is suggested to be based on differences in leaves or other tissue morphological characteristics between species, rather than flowers.

### Variations and evolution of *Pinellia* cp genomes

In this study, the cp genome of *P. pedatisecta* was sequenced (MZ702636) with the length of 164,682 bp, which fell within the cp genome size range for angiosperms but much smaller than its published one (MN046890, 168,178 bp; Table 1). Among four *Pinellia* plastid genomes, LSC regions showed the most difference in size, with the shortest 89,783 bp of *P. ternata* KR270823 and the longest 92,963 bp of *P. pedatisecta* MN046890. Additionally, the inferred structures and protein-coding gene contents were in accordance except for the *infA* and *ycf68* genes (Fig. 1 and S3). Analysis with DnaSP inferred that some of the most divergent regions of *psbI-trnG-UCC, psbM-rpoB, ndhJ-trnT-UGU, trnP-UGG-trnW-CCA, ndhF-trnN-GUU, ndhG-ndhE, ycf1-rps15* and *trnR-ycf1*, as shown in Fig. 2C, were found for further related species identification of *Pinellia*.

SSRs in the cp genome are an efficient marker tool for population genetic structure and phylogeography [47]. In this study, 68–111 SSR loci were identified between *Pinellia* species (Supplementary Table S3). These SSR loci could provide candidate molecular markers for the genetic diversity study of *Pinellia*. The composition of SSR loci in the cp genomes of three *Pinellia* species was similar to that of most angiosperms, with A/T mononucleotide repeats dominating all the repeat units. This phenomenon may be one of the reasons for the abundance of A/T bases in cp genomes. Repeat units in the cp genome can cause sequence polymorphism, providing information for further genetic diversity study of *Pinellia*.

At present, specific studies on the divergence time estimation of *Pinellia* by using molecular data, particularly plastid genomes, are lacking. Li et al. [48] first supposed that species with simple leaves (e.g. *P. peltata*) are less evolved than those with pedate or lobed leaves (e.g. *P. pedatisecta* and *P. ternata*) on the basis of the palynology and isozyme characteristics of *Pinellia* species. Yin [21] speculated that the *Pinellia* genus first appeared in the Paleogene according to geographic distribution and the origin time of its related genus (*Arisaema*). Here, based on whole cp genome sequences, our divergence time estimation indicated that *Pinellia* species originated at ~ 22.48 Mya (95% HPD = 11.39–33.32 Mya; node 3 in Fig. S4; Supplementary Table S8) in the late Oligocene and early Miocene, congruent with that estimated by Yin and further refined, and diverged diversely at ~ 13.09 Mya (95% HPD = 4.91–21.96 Mya; node 4 in Fig. S4) in the Miocene. Furthermore, within the *Pinellia* genus, *P. peltata* diverged first at ~ 5.34 Mya earlier than two other species, *P. pedatisecta* and *P. ternata*; these findings were consistent with the results of Li et al. [48]. The present estimation of divergence times based on plastid genomes and fossil data provides new insights and a hypothetical foundation for future studies on the origin and earlier evolution of *Pinellia*.

**Fig. 4:**
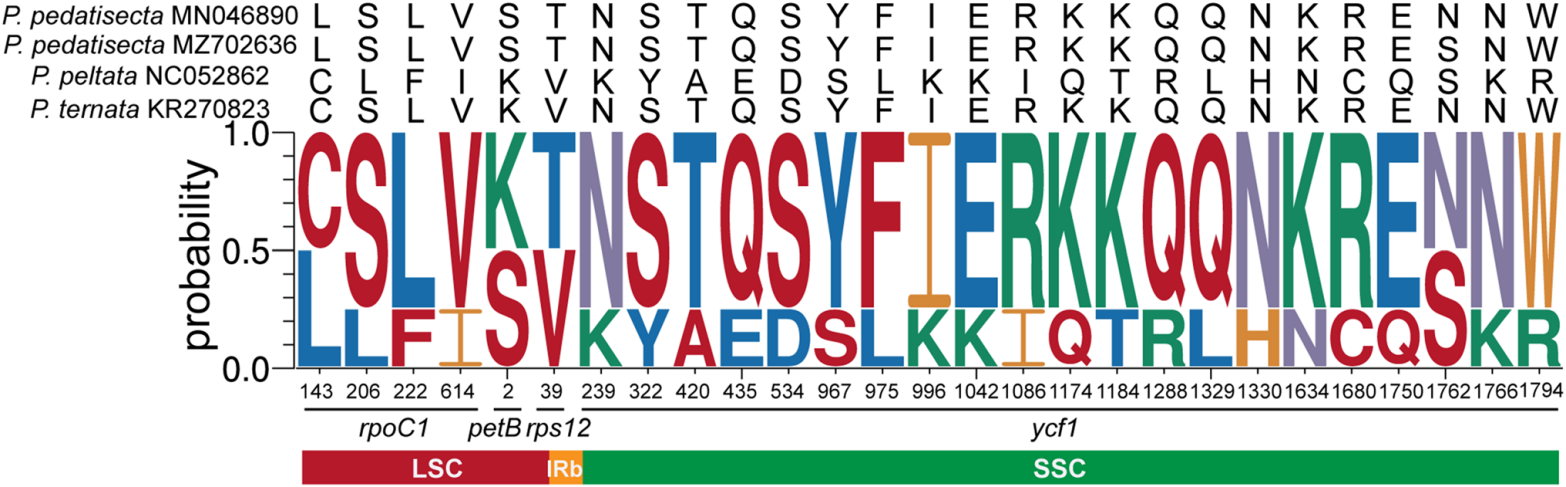
Positive selection sites logo of four *Pinellia* plastomes

Genes subjected to positive selection have a significant impact on the creative effects of populations, changes under selection stress, and genetic drift led to the rapid transformation of genes into new common adaptive combinations [49]. In this study, we calculated the non-synonymous/synonymous substitution rate ratio (*ω* = *dN*/*dS*) for each of the 74 single copy protein-coding genes shared by the analyzed plastid genomes of *Pinellia*. Four genes with high posterior probability of codon sites in the BEB test were acquired and considered as genes under positive selection (Fig. 4 and Tables S9 and S10). These genes included one gene for cytochrome b/f complex subunit protein in the photosystem II reaction (*petB*) [50], one DNA-dependent RNA polymerase gene (*rpoC1*), one gene for ribosome small subunit protein (*rps12*) and *ycf1* gene. All these genes were also detected in other plants [51–54], and these genes may have played a significant role in the adaptive evolution of *Pinellia*. The specific role needs to be further studied.

The genus *Pinellia* includes seven species in total. Only three *Pinellia* species were studied here, and as a consequence, it might not fully show the real effect of the phylogeny of *Pinellia*. However, our study was the first comprehensive phylogenetic analysis of *Pinellia* at the cp genome level, and the resulting topologies based on either whole cp genome or CDS datasets both definitively show that *P. pedatisecta* cannot be the ancestor of *Pinellia* species. The main habitat of all three *Pinellia* species in our study is in the southeastern portion of Asia, where hybridization and introgression have been reported [55, 56], which may cause interspecific gene flow in response to ecological selection and lead to difficulties in molecular phylogenetic analysis. Given that the cp and nuclear genomes evolve independently, the phylogeny of cp genomes alone is insufficient in making taxonomic decisions about *Pinellia*. Therefore, more methods and more species are needed in further study to improve our ability to better understand the phylogeny and evolution of *Pinellia*.

## Conclusions

The genus *Pinellia* Tenore is comprised of seven species, all of which are important medicinal plant resources. Species phylogenetic classification is vital for protecting species diversity and selecting high quality germplasm resources. But due to members of *Pinellia* exhibit rather wide variations in morphological structures, the low-level taxonomy and interspecific phylogeny of *Pinellia* are difficult to address based on morphology. With the development of next-generation sequencing technology, complete chloroplast genomes have been widely employed to explore phylogenetic relationships of intra-or inter-genus. However, the variation and evolution of the whole chloroplast genomes in the genus *Pinellia* have been ignored. Here, we sequenced the chloroplast genome of *P. pedatisecta*, compared it with previously published plastid genomes, and reconstructed phylogenetic relationships of *Pinellia* based on chloroplast genomes and their CDSs. The divergence time estimation and selective pressure of *Pinellia* plastomes were also investigated. The results showed some variations and adaptive evolution between *Pinellia* complete chloroplast genomes including size, structure and nucleotide diversity, which provided valuable information for species classification and evolution. In addition, our results revealed a sister relationship between *Pinellia* and *Arisaema* plants whose divergence was estimated to occur around 22.48 million years ago. All *Pinellia* species formed a monophyletic evolutionary clade in which *P. peltata*, rather than *P. pedatisecta*, earlier diverged, indicating that *P. pedatisecta* is not the basal taxon of *Pinellia* but *P. peltata* may be. In conclusion, our results could provide insight into the chloroplast genome evolution and phylogeny of *Pinellia* genus and even of Araceae family, which would be useful for selecting high quality *Pinellia* germplasm resources in the future.

## Supplementary Information

Below is the link to the electronic supplementary material.

(DOCX 2878 kb)

(XLSX 148 kb)

## Data Availability

The chloroplast genome sequence of *Pinellia pedatisecta* assembled here is accessible via GenBank with the accession number of MZ702636 and Global Pharmacopoeia Genome Database (GPGD, http://www.gpgenome.com/species/40314). Raw sequencing data is available at NCBI SRA database with the accession number of SRR15328795.

## Abbreviations

cp: Chloroplast
SSRs: Simple sequence repeats
WGS: Whole-genome sequencing
PE: Paired-end
BLAST: Basic Local Alignment Search Tool
LSC: Large single copy
SSC: Small single copy
IR: Inverted repeat
indel: Insertion-deletion
Pi: Nucleotide diversity
CDS: Protein-coding sequence
ML: Maximum likelihood
Mya: Million years ago
ESS: Effective sample size
LRT: Likelihood ratio test
BEB: Bayes Empirical Bayes approach
HPD: Highest posterior density
MP: Maximum parsimony
LCBs: Locally collinear blocks
