# Supplementary material for: Comparative chloroplast genomes and phylogenetic analyses of Pinellia
Source: Mol Biol Rep. 2022 Jun 11;49(8):7873–85. doi: 10.1007/s11033-022-07617-5 (PMC9304046; doi:10.1007/s11033-022-07617-5)
Supplement: Supplementary file 1 — (DOCX 2878 kb) [file 11033_2022_7617_MOESM1_ESM.docx]

**Title: Comparative chloroplast genomes and phylogenetic analyses of *Pinellia***

Authors: Ning Cui^1^·Weixu Chen^2^·Xiwen Li^3^·Ping Wang^1^

^1^ Central Laboratory, Shandong Academy of Chinese Medicine, Ji’nan, China

^2^ Shang yao hua yu (LinYi) Traditional Chinese Medicine Resources Co., Ltd., Pingyi, China

^3^ Institute of Chinese Materia Medica, China Academy of Chinese Medical Sciences, Beijing, China

**Supplementary Figure legends**

**Fig. S1** The phylogenetic analyses of *Pinellia* genus with MP method by using ITS (A), *matK* (B), *rbcL* (C), and *trnL-F* (D) sequences downloaded from NCBI database. Node support is given as bootstrap values (1000 bootstrap replicates).

**Fig. S2** The gel picture of PCR products for verifying the assembly of *P. pedatisecta* cp genome. 1. PCR product using the primer of LSC/IRb junction. 2. PCR product using the primer of IRb/SSC junction. 3. PCR product using the primer of SSC/IRa junction. 4. PCR product using the primer of IRa/LSC junction. 5. PCR product for checking the indel variation between published and self-assembled *P. pedatisecta* cp genomes. Marker: DL 2,000 DNA Marker (Takara Bio).

**Fig. S3** Comparison of the cp genome gene content of Araceae plants and one model plant (*Arabidopsis thaliana*). Only protein-coding genes with different numbers in 25 cp genomes are listed. Red boxes indicate two copies of each gene, and yellow boxes indicate a single copy of each gene. The blue boxes indicate an absent gene.

**Fig. S4** Chronogram showing divergence times estimated based on 27 whole chloroplast cp genomes. Divergence time are shown for each node. Purple bars represented 95% HPD values for the estimated mean dates. Nodes labelled red pentagram were calibration points used for analysis. Numbers 1-4 indicated nodes of interest.

**Fig. S5** Gene order comparison of four plastid genomes of *Pinellia*. Alignment and resulting locally collinear blocks (LCBs) were generated using MAUVE. (A) A comparison of four complete cp genomes of *Pinellia*. (B) The local magnification of the gene traslocation between the chloroplast genomes of *P. pedatisecta* MN046890 and *P. ternata* KR270823. (C) The start position of the large insertion-deletion (indel) variation across four *Pinellia* plastid genomes. (D) The end position of this indel variation of four cp genomes.


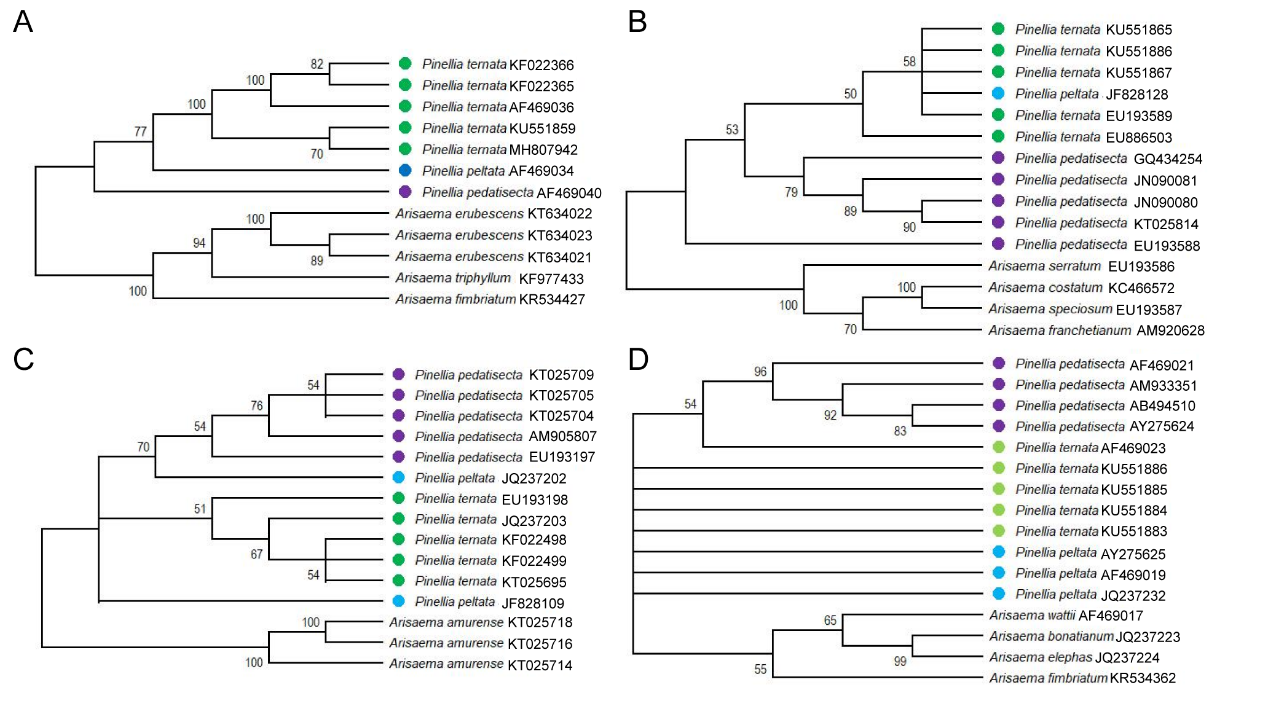


**Fig. S1** The phylogenetic analyses of *Pinellia* genus with MP method by using ITS (A), *matK* (B), *rbcL* (C), and *trnL-F* (D) sequences downloaded from NCBI database. Node support is given as bootstrap values (1000 bootstrap replicates).


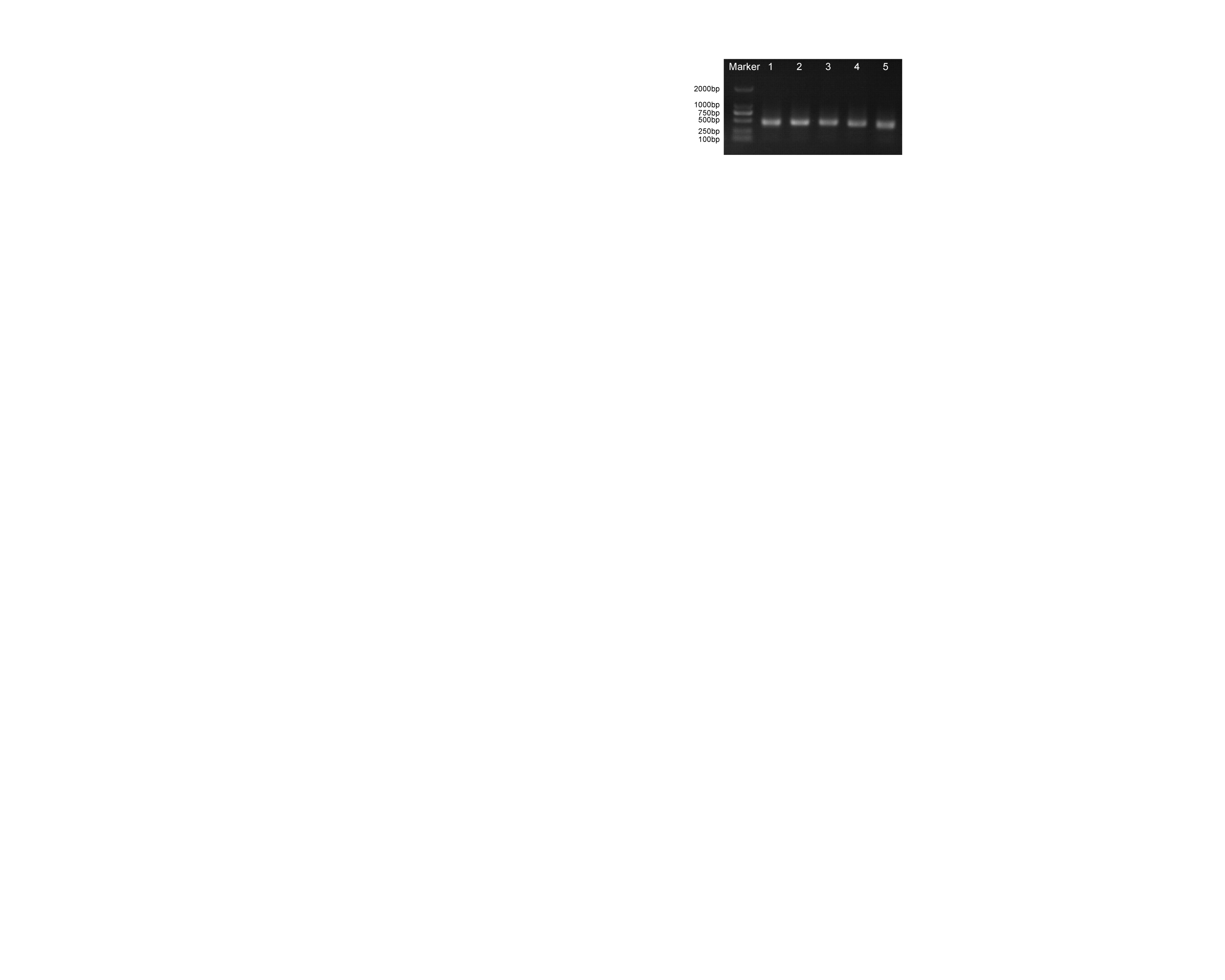


**Fig. S2** The gel picture of PCR products for verifying the assembly of *P. pedatisecta* cp genome. 1. PCR product using the primer of LSC/IRb junction. 2. PCR product using the primer of IRb/SSC junction. 3. PCR product using the primer of SSC/IRa junction. 4. PCR product using the primer of IRa/LSC junction. 5. PCR product for checking the indel variation between published and self-assembled *P. pedatisecta* cp genomes. Marker: DL 2,000 DNA Marker (Takara Bio).


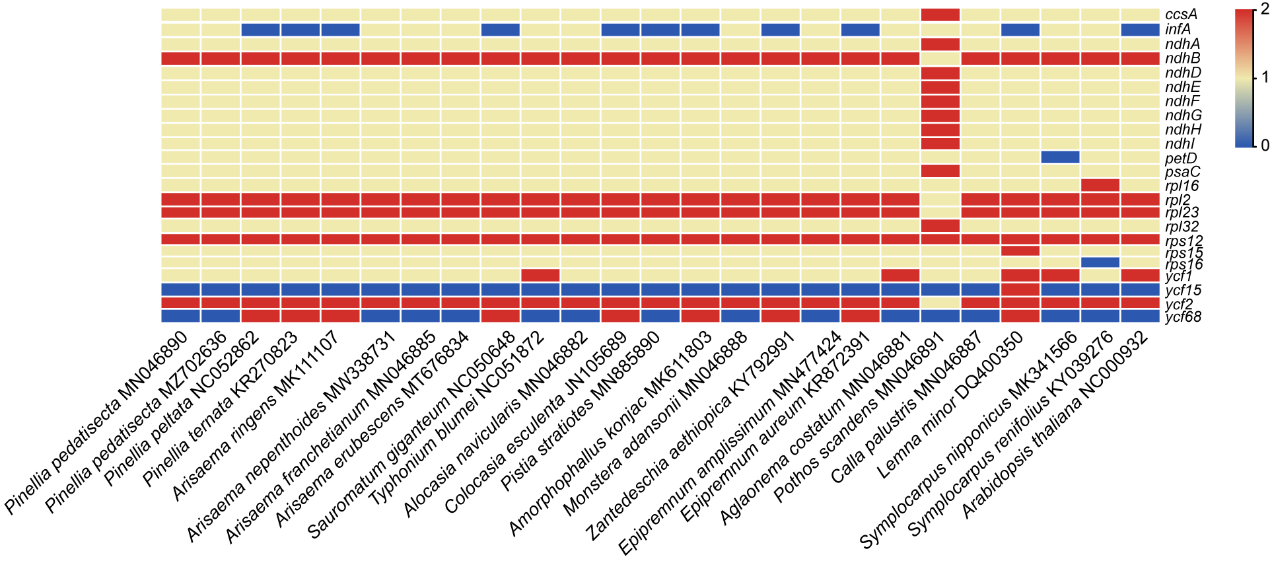


**Fig. S3** Comparison of the cp genome gene content of Araceae plants and one model plant (*Arabidopsis thaliana*). Only protein-coding genes with different numbers in 25 cp genomes are listed. Red boxes indicate two copies of each gene, and yellow boxes indicate a single copy of each gene. The blue boxes indicate an absent gene.

**
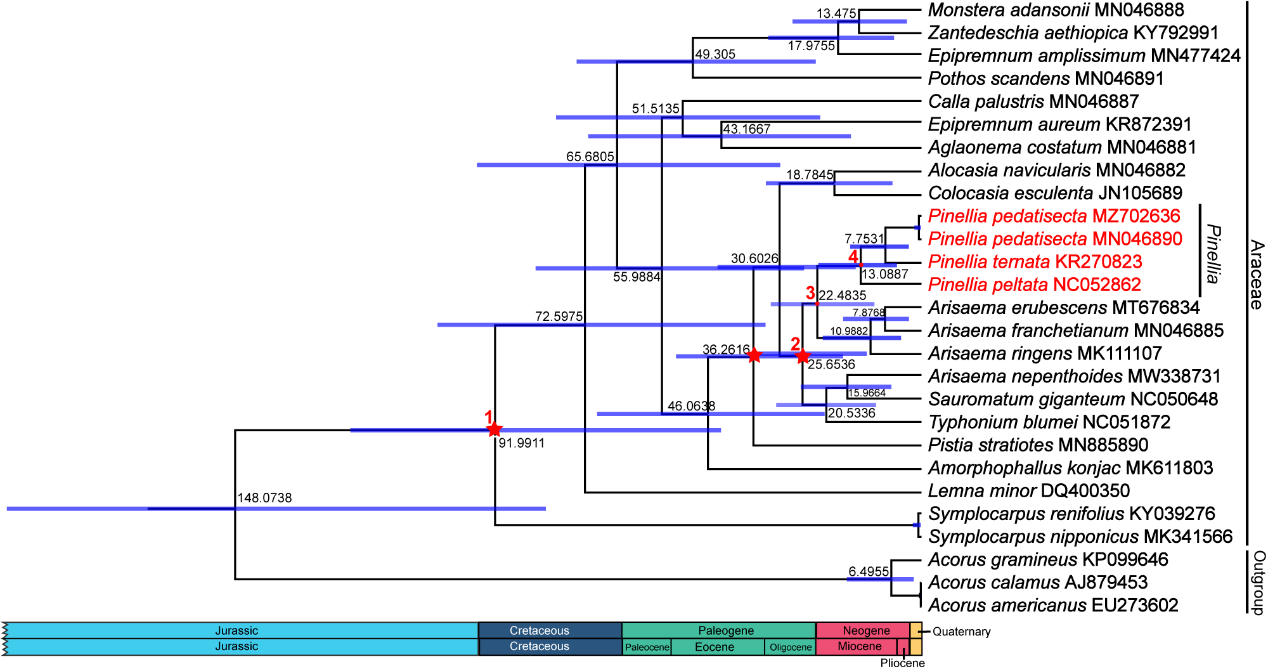
**

**Fig. S4** Chronogram showing divergence times estimated based on 27 whole chloroplast cp genomes. Divergence time are shown for each node. Purple bars represented 95% HPD values for the estimated mean dates. Nodes labelled red pentagram were calibration points used for analysis. Numbers 1-4 indicated nodes of interest.


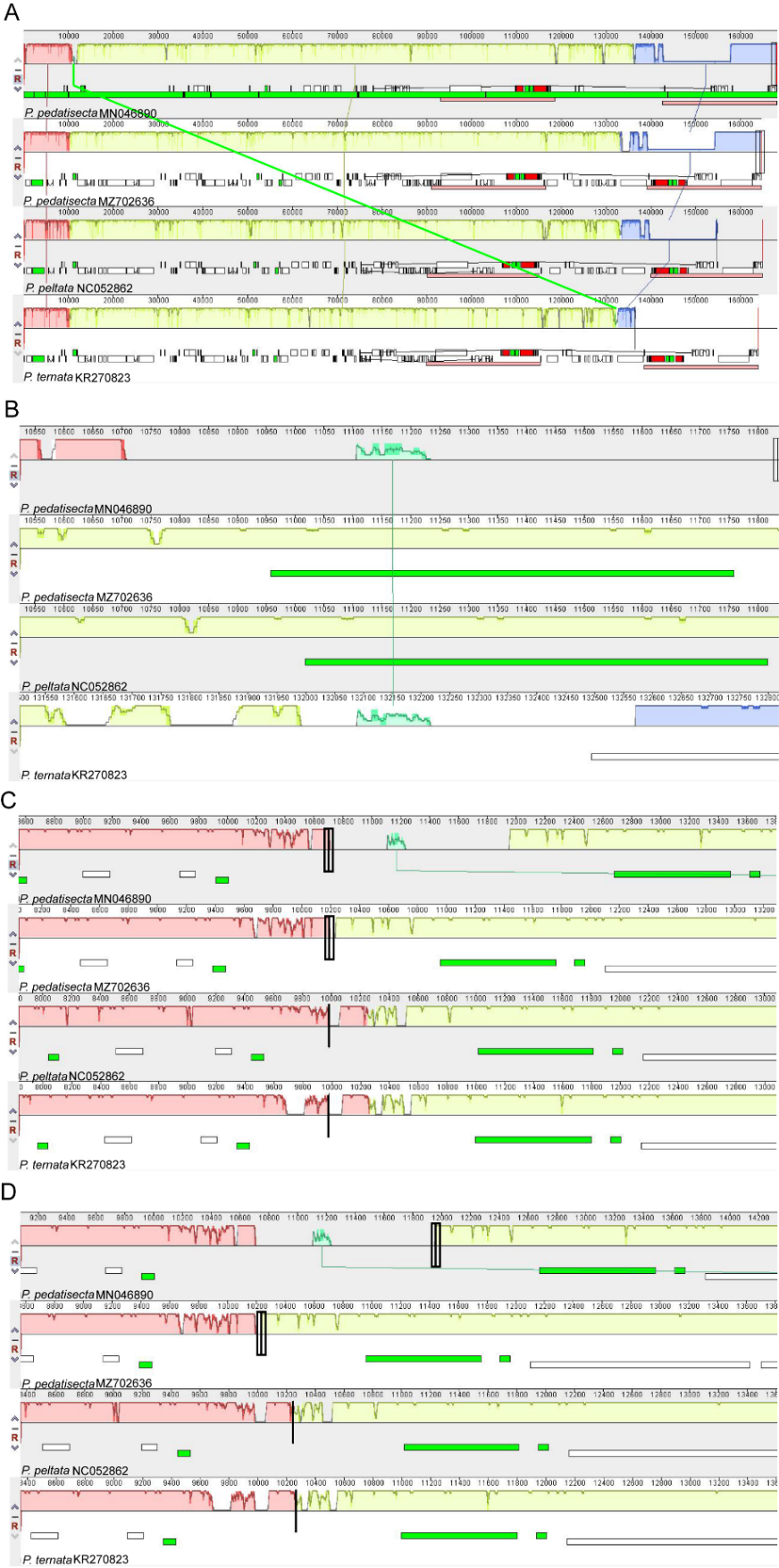


**Fig. S5** Gene order comparison of four plastid genomes of *Pinellia*. Alignment and resulting locally collinear blocks (LCBs) were generated using MAUVE. (A) A comparison of four complete cp genomes of *Pinellia*. (B) The local magnification of the gene traslocation between the chloroplast genomes of *P. pedatisecta* MN046890 and *P. ternata* KR270823. (C) The start position of the large insertion-deletion (indel) variation across four *Pinellia* plastid genomes. (D) The end position of this indel variation of four cp genomes.
